# Can Severe Uremia Impact Mortality Predictors in Elderly People With Kidney Failure?

**DOI:** 10.7759/cureus.72715

**Published:** 2024-10-30

**Authors:** Gvantsa Metskhvarishvili, Gaiane Simonia, Nora Sarishvili, Irma Tchokhonelidze

**Affiliations:** 1 Internal Medicine, Tbilisi State Medical University, Tbilisi, GEO; 2 Nephrology, Tbilisi State Medical University (TSMU) and Ingorokva High Medical Technology (HMT) University Clinic, Tbilisi, GEO

**Keywords:** chronic kidney disease, elderly patients, frailty, mortality predictor scores, unplanned dialysis

## Abstract

Numerous studies have shown that dialysis may not be as beneficial to elderly, frail patients with chronic kidney failure and multiple comorbidities as comprehensive conservative therapy (CCT) and that dialysis may worsen the quality of life (QOL), increase hospitalization rates, and cause a significant decline in functional status. Several mortality predictors have been proposed to determine which patients would benefit more from CCT or dialysis.

We estimated the short-term risk of death in an 81-year-old male patient with kidney failure and highly severe frailty using the REIN score, a dependable risk prediction model proposed by the European Renal Best Practice Group for the prediction of short-term risk mortality. This score indicated that the patient had a high chance of death in the ensuing three months. However, the patient’s longer survival time and a notable increase in functional status and QOL following hemodialysis started to contradict the expected outcome. It is important to note that the patient had never undergone a frailty assessment before or had been on a nephrologist’s follow-up.

We suggest that uremia may exaggerate frailty levels in older persons and as a result, undermine the predictive usefulness of mortality prediction scores in this population.

## Introduction

Making the decision to start dialysis for elderly, frail patients with chronic kidney disease (CKD) is seen as difficult for medical professionals, patients, and the patient’s family or caregivers. Therefore, while providing treatment choices and counseling elderly patients with kidney failure (KF), it is essential to have reliable prognostic techniques to determine an individual’s life expectancy within a particular time frame, both with and without dialysis initiation. The European Renal Best Practice Group’s guidelines for the management of elderly patients with CKD stage 3b or higher [1] recommend using the Bansal score for 5-year mortality prediction [2] and the REIN score for short-term mortality risk prognosis in patients aged 65 years and older [3,4]. Nevertheless, research from Romania and Spain revealed inconsistent findings, citing little predictive value for Bansal and REIN scores, respectively [5,6].

## Case presentation

A case study of an 81-year-old male patient is presented herein, whose family was faced with the decision of whether to initiate dialysis or to continue comprehensive conservative therapy (CCT) [7] following the patient’s acute kidney injury recovery that developed on CKD. Written informed consent was obtained from the patient for publication of this case.

The patient was diagnosed with CKD in 2019, but he had not since seen an outpatient nephrologist. As a result, there were no records of the patient’s mental state, degree of CKD progression, or frailty status [8]. He had very severe frailty (assessed by the Canadian Study in Health and Aging (CSHA) Clinical Frailty Scale (CFS)) [9], heart failure (ejection fraction (EF) 45%), and delirium at the time of unscheduled hospital admission. Consequently, the patient could not participate in the final shared decision-making process and did not understand the management of CKD and the available modalities.

His medical records revealed that he had been diagnosed with prostate cancer diagnosis 10 years ago, but he had also neglected to undergo oncology examinations. Findings from the lab studies included Crea-473 µmol/L eGFR-Crea, Cystatin C-9 mL/min/1.73 m^2^, Alb-27 g/L, Hb-9.9 g/dL, PH-7.4, HCO_3_-24 mmol/L, and K-3.5 mmol/L. The family was advised of the significant chance of death due to the patient’s advanced age, frailty, and comorbidities. The Bansal score (12ps) [1], clinical frailty scale (9), and the REIN score (20ps) [3,4] were used to predict the 5-year and 3-month mortality risks, respectively (Tables 1-2). Deciding between kidney replacement therapy (KRT) and CCT was challenging.

**Table 1:** Bansal Score eGfr-Crea mL/min/1.73 m^2^; eEGFR: estimated glomerular filtration rate mL/min/1.73 m^2^; urine A/C ratio: urine albumin/creatinine ratio

Bansal Score	12 points
Age	2 points
Sex	1 point
Race	1 point
eGfr-Crea mL/min/1.73m^2^	4 points
Urine A/C ratio>30 mg/g	1 point
Diabetes	0 point
Smoking	1 point
Prevalent heart failure	2 points
Prevalent stroke	0 point

**Table 2:** REIN Score

REIN Score results	20 points
Gender	1 point
Age	1 point
Congestive heart failure	2 points
Peripheral vascular disease	0 point
Dysrhythmia	0 point
Cancer	2 points
Severe behavior disorder	2 points
Mobility	9 points
Albumin	3 points
Chronic respiratory disease	0 point

The patient’s relatives considered moving the patient to a palliative care facility and made multiple changes to their original decision. Finally, the patient resumed hemodialysis (HD). His schedule was limited to twice a week. At that point, the patient was completely reliant on the caregivers and required a wheelchair as well as the assistance of two or more family members to get to the HD unit. However, the patient’s overall condition and quality of life (QOL) significantly improved three months after starting dialysis; the patient’s kidney disease quality of life (KDQOL) overall health component scores increased from 30 to 60 points; the KDQOL energy/fatigue component increased tenfold from 5 to 50; the KDQOL physical functioning component increased from 0 to 25; the KDQOL emotional well-being component increased from 12 to 72; the cardiovascular status (CV) also improved (EF increased from 45% to 52%) and the severity of frailty decreased (from CFS 8 to 4) (Table 3).

**Table 3:** Follow-up data of the patient *HD: hemodialysis **KDQOL: Kidney Disease Quality of life; KDQOL-SF™ Version 1.3 Scoring Program used for KDQOL assessment (0–100 possible scores). ***Rockwood Clinical Frailty Scale (1- Very fit, 2- well, 3- Managing well, 4- Vulnerable, 5-Mildly frail, 6- Moderately frail, 7- Severely frail, 8- Very severely frail 9- Terminally ill)

Follow-up data	Before HD*	3 months after starting HD	6 months after starting HD	1 year after starting HD	2 years after starting HD
Albumin (Reference range: 35-55 g/L)	25 g/L	45 g/L	41 g/L	37 g/L	38 g/L
Ejection fraction (Reference range: 55-70%)	45%	52%	52%	52%	52%
KDQOL cognitive function^**^	33	80	100	100	100
KDQOL overall health^**^	30	60	70	70	70
KDQOL mental composite^**^	25.22	58.6	59.35	56.40	58.97
KDQOL physical composite^**^	33.49	29.27	37.3	49.85	47.6
KDQOL energy/fatigue^**^	5	50	80	80	75
KDQOL physical functioning^**^	0	25	55	75	80
KDQOL general health^**^	00	25	35	50	45
KDQOL emotional well-being^**^	12	72	76	76	88
Hospitalization rate	1 in 30 days before HD	0	0	0	1
Clinical frailty scale***	8	6	5	4	4

Six months later, the patient’s condition improved even further; he regained independence, could visit the dialysis facility without help or carers, and was actively involved in the winemaking process at his family winery. Two years after beginning KRT, the patient is still on HD twice a week, has only required one hospital stay for HD catheter-related infection in two years since starting KRT, and can retain the same QOL.

## Discussion

This case highlights the difficulty in selecting the best course of treatment for older people with CKD. In particular circumstances, even the most credible prognostic scores might not be able to accurately forecast the outcome, especially in cases of unexpected dialysis where the history of frailty assessment is undetermined. Our patient was assigned to the high-risk mortality group within three months based on the REIN score of 20 points. Nonetheless, his survival was longer than expected. Furthermore, at 3-6 months after beginning KRT, the patient’s emotional well-being, frailty score, QOL, and CV status greatly improved. This underlines the strong suspicion that frailty assessment before severe uremia is essential for predictive scores. In the uremic state, frailty is mostly influenced by uremic toxins and does not reflect the patient's actual state based on age or comorbidities [10,11].

Predicting morbidity and mortality events more precisely is possible if frailty is identified prior to the start of dialysis. In particular, when it comes to the unplanned onset of dialysis, which occurs between 40% and 60% of the time [12]. Therefore, if it is the patient’s first assessment in a long time, as in the case above, the predictive value of the mortality predictive scores may be compromised, and the benefit from KRT may be underestimated if one relies solely on the mortality predictor score and ignores severe uremia. More studies are required to fully comprehend the potential benefits of KRT in cases of advanced CKD when frailty has not been previously assessed promptly or regularly. This could be accomplished by reassessing frailty and cognitive status three to six months after the beginning of KRT.

## Conclusions

This case illustrates the challenge of finding the best course of action for elderly individuals who have CKD. In certain circumstances, even the most reliable prognostic scores may not be able to predict the outcome, especially in cases of unexpected dialysis when the past history of frailty assessment is unclear. We hypothesize that elderly individuals with uremia may become more fragile, potentially decreasing the predictive value of mortality prediction scores in this population. Furthermore, the predictive value of the mortality prediction scores may be compromised if this is a patient's first assessment and there is no dynamic description of frailty status before. Neglecting the impact of severe uremia on a patient’s cognitive and physical condition may underestimate the beneficial effect of KRT. The patient requires a re-assessment shortly after starting dialysis treatment.
